# Hemolytic Transfusion Reaction Due to Anti-A_1_ Antibody During Pregnancy: Case Report

**DOI:** 10.1155/crii/7705026

**Published:** 2025-11-06

**Authors:** Suhalika Sahni, Katharine Sweeney, Franklin Njoku, David Allison

**Affiliations:** ^1^Department of Pathology, University of Illinois at Chicago, Chicago, Illinois, USA; ^2^Department of Obstetrics and Gynecology, University of Illinois at Chicago, Chicago, Illinois, USA; ^3^Department of Medicine, University of Illinois at Chicago, Chicago, Illinois, USA

**Keywords:** hemolytic transfusion reaction, hyperhemolytic syndrome, sickle cell disease

## Abstract

The ABO blood group is the most clinically relevant system in transfusion medicine. Approximately 20% of individuals with blood group A of European descent belong to a weak A subgroup, most commonly A_2_, which may produce anti-A_1_ antibodies. These antibodies are usually cold-reactive IgM and rarely cause hemolysis, but can occasionally be clinically significant when reactive at 37°C. We describe a pregnant woman with sickle cell disease (HbS/β^0^ thalassemia) and prior hyperhemolysis syndrome who developed a severe delayed hemolytic transfusion reaction (DHTR) after transfusion of A_1_ red blood cells (RBCs). Anti-A_1_ was identified posttransfusion, confirming her as a non-A_1_ subtype. Notably, she also experienced hemolysis following group O red cell transfusion, consistent with hyperhemolysis. This case highlights the rare but serious potential of anti-A_1_ to cause DHTR, particularly in high-risk populations, and underscores the importance of increased vigilance when managing transfusion in sickle cell disease.

## 1. Introduction

The ABO blood group is the only blood group in which individuals form naturally occurring antibodies (isohemagglutinins) to the antigens absent from their red blood cells (RBCs) without a previous sensitizing event such as prior transfusion [1, 2]. In blood groups A and B, these antibodies are primarily IgM with lesser amounts of IgG; in group O, however, IgG is the primary isotype. IgM agglutinates reagent RBCs at room temperature (cold antibody) and activates the complement cascade, which can cause severe hemolytic transfusion reactions (HTRs) that may result from ABO-incompatible transfusion [2, 3].

Infants begin producing isohemagglutinins, or isoagglutinins, at 4–6 months of age and peak by 10 years [2, 3]. By test design, ABO antibodies produce strong agglutination reactions (at least 3+ reactivity) during ABO typing. The ABO system contains several weak subtypes, amongst which A_2_ is the most prevalent [4]. Serologic clues to suggest an A-subtype include: (1) strong agglutination of a patient's RBCs with anti-A antisera (forward type) combined with unexpected reactivity against reagent A_1_ cells and the patient's plasma (reverse type), or (2) unexpectedly weak forward type reactions (less than 3+ strength). A_2_ RBCs may be serologically distinguished from A_1_ RBCs by their lack of reactivity with the lectin *Dolichos biflorus*, which agglutinates *A*_1_ but no other A subtypes [2, 5]. These weak phenotypes, in most cases, result from the expression of a variant A allele present at the ABO loci [1]. The primary risk for an individual with an A subtype is having a serious HTR following transfusion of a group A RBC unit if the patient produces anti-A_1_. The risk of anti-A_1_ causing RBC hemolysis appears to be higher if the antibody causes agglutination at 37°C (warm-reacting antibody) and lower if the antibody does not cause agglutination at 37°C (cold-reacting antibody).

All ABO discrepancies between the forward type and reverse type of patient samples must be resolved prior to issuing blood products. If transfusion is necessary before resolution, or results cannot be resolved, group O RBCs and group AB plasma may be issued according to institutional emergency release procedures [3].

Anti-A_1_ antibodies are typically low-titer, cold-reactive, and nonreactive at 37 °C, and for this reason, they are generally regarded as clinically insignificant. The few reports in the literature describing clinically significant anti-A_1_ either present an immediate reaction to a preformed high-titer, warm-reactive anti-A_1_ (boosted by previous transfusion with group A_1_ blood), or a very mild delayed reaction [6]. We present the case of a woman with a previously undiagnosed group A subtype with clinically significant anti-A_1_ who experienced a serious HTR following transfusion of group A_1_ RBCs.

## 2. Case Presentation

A 30-year-old female patient, blood group A Rh positive and with a negative antibody screen, presented with a history of sickle cell disease (HbS/beta-zero thalassemia) and hyperhemolytic syndrome (HS), which she experienced approximately 10 years earlier. She was first admitted during her pregnancy at 10 weeks of gestational age (GA) due to dyspnea and fatigue, where she was treated for acute chest syndrome (ACS). At this visit, her hemoglobin (Hb) had dropped to a low of 5.8 g/dL. As part of her treatment, she received one transfusion of group A Rh-negative RBCs. This was done following a standard premedication regimen, which included methylprednisolone for 2 days and intravenous immunoglobulin (IVIG) for 5 days, due to her known history of HS.

She returned at 30 weeks and 6 days GA with a repeat presentation for ACS and an Hb nadir of 6.3 g/dL. She was premedicated with IVIG and methylprednisolone and received two units of group O RBCs. Her Hb peaked at 8.3 g/dL on Hospital Day 6, and she was subsequently discharged.

Twenty days later, she was admitted to our facility at 34 weeks and 1 day GA for a headache and a drop in Hb levels from 8.3 to 6.8 g/dL, with a subsequent nadir of 6 g/dL. Hb electrophoresis showed undetectable levels of HbA, despite recent transfusions during her prior hospitalization. Both her antibody screen and direct antiglobulin test (DAT) returned negative results. She remained hospitalized for treatment, which included erythropoietin-stimulating agent, folic acid, and iron supplementation. Additionally, she received IVIG and methylprednisolone for a delayed HTR (DHTR).

The patient was readmitted to our facility at 36 weeks and 5 days GA for induction of labor. She was premedicated in preparation for RBC transfusion. Two days after her admission, and prior to labor, her Hb decreased to 6.1 g/dL, prompting the administration of two units of group A RBCs between hospital Days 3 and 4. Following the transfusion, her Hb improved to 8.6 g/dL.

**Figure 1:**
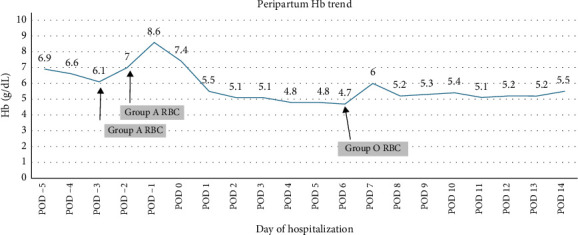
Hemoglobin trends during the hospitalization for cesarean delivery. Arrows indicate RBC transfusions. Image legend: POD— post operative day.

She underwent an urgent repeat low transverse cesarean delivery due to category II fetal tracings. The total blood loss during the procedure was 675 mL. On post-op day 1 (POD 1), a repeat type and screen demonstrated an ABO discrepancy (Table 1). The antibody screen was positive. Polyspecific DAT and IgG monospecific DAT were positive, and C3 monospecific DAT was negative. Anti-A_1_ was identified in the eluate. By POD 2, the patient's Hb had decreased to 5.1 g/dL and eventually dropped to a nadir of 4.7 g/dL before slowly recovering (Figure 1).

The patient was phenotyped, and her RBCs were negative for the A_1_ antigen, confirming her as an A-subtype. She was placed on an O RBC protocol. She received one group O-Rh negative RBC in response to her Hb of 4.7 g/dL, which was well-tolerated, and she was discharged 1 week later with Hb 5.5 g/dL.

## 3. Discussion

This case demonstrates the complex interplay of alloimmunization, weak ABO subtypes, and transfusion complications in a high-risk patient with sickle cell disease. The patient experienced two distinct hemolytic events during her course. The first, a DHTR to group A_1_ red cell units. The second episode, which followed transfusion of group O units, was characterized by a precipitous Hb decline and undetectable HbA despite the absence of detectable alloantibodies and a negative DAT.

DHTR was confirmed by the development of a positive antibody screen and DAT with eluate identification of anti-A_1_. Although anti-A_1_ antibodies are generally low-titer, cold-reactive, and rarely of clinical significance, in this instance, the antibody was reactive at 37°C and clearly contributed to a severe and clinically meaningful hemolytic reaction. The second episode is most consistent with HS, a rare but recognized transfusion reaction in SCD, where both autologous and transfused RBCs are destroyed. Although group O transfusions should have been compatible with the patient's A subtype, the clinical course reflected an immune-mediated clearance process consistent with her prior history of HS.

HTRs are significant causes of transfusion-related morbidity. In a 2015 FDA report, 21% of transfusion fatalities were caused by HTRs; 7.5% were due to ABO incompatibility [7]. DHTRs can occur from 1 to 28 days after RBC transfusion [8]. These reactions can occur in patients who have been alloimmunized to minor RBC antigens during previous transfusions or pregnancies when subsequent pretransfusion testing fails to detect these alloantibodies in patient plasma [9]. Investigation of HTR includes checking for clerical errors during issuance or transfusion, DAT, visual hemolysis screen (plasma and urine), lactate dehydrogenase, indirect bilirubin, and haptoglobin. In patients with SCD, a posttransfusion Hb electrophoresis and absolute reticulocyte counts are useful [9, 10]. Zerra and Josephson [11] previously described the procedure to resolve a positive antibody screen or a positive DAT and the elution process to identify or exclude any clinically significant alloantibodies.

Weak agglutination reactions can arise from a low expression of A and B antigens on the surface of RBCs, potentially leading to discrepancies in blood group typing. Due to variations in reagents and methods, these weaker phenotypes may be incorrectly classified as blood type O. In rare cases, weaker subgroups of A can create practical issues; for example, an A-subtype donor may be misidentified as group O and, as a result, transfused to a true group O recipient [2]. This is potentially dangerous because the group O recipient possesses antiA, B which could lyse donor RBCs of group A-subtype, causing hemolysis of the transfused RBCs. Accurate ABO-subtyping may also prevent unnecessary transfusion of “O” RBCs and “AB” plasma, better conserving these critical components [1]. Genotyping can be performed to resolve ABO typing discrepancies, and methods include different types of polymerase chain reaction (PCR), such as restriction fragment length polymorphism (PCR-RFLP) or polymerase chain reaction-sequence-specific primers (PCR-SSP) [12]. Adoption of routine ABO genotyping for transfusion purposes varies widely across institutions. Although routine genotyping for all transfusion recipients is unlikely to be practical at present, targeted application in patients at risk of severe hemolytic complications could be a valuable safety measure. Often, with serologic ABO discrepancies, the most practical and conservative strategy is simply assigning the patient to receive group O RBCs and AB plasma.

Regardless of the exact pathophysiologic mechanism for our case's hemolytic reactions, the management is similar. In 2020, the American Society of Hematology (ASH) published transfusion guidelines for sickle cell disease [13]. This guidance paper provides recommendations for managing patients with SCD who are at high risk of acute hemolytic reactions or who have a history of multiple or serious DHTRs. The panel recommended administering immunosuppressive agents before transfusions. They also suggested using treatments like IVIG, steroids, rituximab, or eculizumab for patients experiencing DHTR or hyperhemolysis.

This report is limited by the lack of molecular confirmation of the patient's A subtype, as our reference laboratory does not perform routine genotyping. In addition, as a single case report, the findings cannot be generalized. Nonetheless, the severe clinical course highlights the need for greater awareness of anti-A_1_ as a potential cause of HTR. Ultimately, the management of this patient required not only accurate recognition of the serologic discrepancy but also adaptation of future transfusion strategy to prevent recurrence. Greater recognition of the potential significance of anti-A_1_, combined with individualized transfusion planning and consideration of conservative approaches such as O-unit transfusion in unresolved cases, may improve safety for similarly vulnerable patients.

## Figures and Tables

**Table 1:** Forward and reverse typing discrepancy, showing 2+ reactivity between the patient's plasma and A_1_ cells.

Reagent and reaction strength	Forward type			Reverse type	
Reagent	Anti-A	Anti-B	Anti-D	A_1_ Cells	B Cells
Reaction	4+	0	4+	2+	4+

## Data Availability

The data sharing is not applicable to this article as no new data were created or analyzed in this study.
